# Internet-Based Cognitive Behavioral Therapy for Children and Adolescents With Dental or Injection Phobia: 1-year Follow-Up Assessment

**DOI:** 10.2196/80376

**Published:** 2025-09-17

**Authors:** Robert Schibbye, Erik Hedman-Lagerlöf, Viktor Kaldo, Göran Dahllöf, Shervin Shahnavaz

**Affiliations:** 1 Division of Pediatric Dentistry Department of Dental Medicine Karolinska Institutet Huddinge Sweden; 2 Center of Pediatric Oral Health Stockholm Sweden; 3 Division of Psychology Department of Clinical Neuroscience Karolinska Institutet Stockholm Sweden; 4 Centre for Psychiatry Research Department of Clinical Neuroscience Karolinska Institutet, & Stockholm Health Care Services Region Stockholm Sweden; 5 Department of Psychology Faculty of Health and Life Sciences Linnaeus University Växjö Sweden; 6 Center for Oral Health Services and Research Mid-Norway (TkMidt) Trondheim Norway

**Keywords:** adolescents, CBT, cognitive behavioral therapy, ICBT: internet-based cognitive behavioral therapy, dental fear and anxiety, children, dental anxiety, dental fear, dental phobia, injection phobia, internet, specific phobia

## Abstract

**Background:**

Dental phobia (DP) and injection phobia (IP) are common in pediatric populations, resulting in inability to receive dental care. Internet-based cognitive behavioral therapy (ICBT) has demonstrated efficacy, but its long-term effects are unexplored.

**Objective:**

This study aimed to evaluate the long-term effects of ICBT on DP and IP in children and adolescents.

**Methods:**

In total, 49 participants (mean age 11.1 years, SD 2.1) with DP, IP, or both underwent a 12-week, parent-guided, exposure-based ICBT, supplemented by visits at local dental clinics and weekly psychologist correspondence. Assessments occurred at baseline, posttreatment, and 1-year follow-up. Primary outcomes included diagnostic status (clinical interview) and ability to receive dental procedures. Secondary outcomes included measures of dental anxiety, injection anxiety, negative cognitions, and self-efficacy. The study was conducted in Sweden.

**Results:**

Of the 49 participants, 42 (86%) completed the 1-year follow-up. At 1-year follow-up, 19 (53%) of 36 (86%) participants who initially met the criteria for DP no longer did (*P*<.001), and 17 (46%) of 37 (88%) participants who initially met the criteria for IP did not fulfill the IP diagnosis (*P*<.001). Repeated-measures ANOVA showed significant improvements, with large effect sizes for self-reported ability to undergo dental procedures (d=1.1, *P*<.001), dental fear (d=1.0, *P*<.001), negative cognitions (d=0.9, *P*<.001), injection fear (d=0.7, *P*<.001), and self-efficacy (d=1.1, *P*<.001). Predictor analysis showed greater improvements in older participants and males.

**Conclusions:**

This study discussed the clinical implications of and approaches to ICBT implementation. ICBT for children and adolescents with DP and IP maintains its effects over a 1-year follow-up period, facilitating improved self-reported willingness to undergo dental treatment. Given its accessibility and sustained efficacy, ICBT should be considered for managing severe dental fear in pediatric dentistry.

**Trial Registration:**

ClinicalTrials.gov NCT02588079; https://clinicaltrials.gov/study/NCT02588079

## Introduction

Dental fear and anxiety (DFA) is widespread, affecting approximately 24% of all children and adolescents [1]. When fear becomes so intense that it significantly disrupts an individual’s functioning, it can be classified as a specific phobia according to the *Diagnostic and Statistical Manual of Mental Disorders, Fifth Edition* (DSM-5) [2]. A phobic response involves avoiding the feared object; in the context of dental phobia (DP), this often results in patients greatly delaying or being unable to seek dental care [3]. Failing to attend or delaying visits to the dentist is associated with poor oral health [4], making DP a serious health concern for those affected.

Pediatric dentistry has methods for preventing fears and providing children with DFA treatment, including pharmacological treatments and dental behavior support (DBS) [5]. However, the DBS conventionally used has not proven to be as effective in treating children with DP [6]. Similarly, the evidence regarding pharmacological sedation to reduce anxiety is conflicting and inconclusive, with no reports on the long-term effects of reduced anxiety [7].

In contrast, cognitive behavioral therapy (CBT) has been shown to be effective in both children [8] and adults with high DFA or DP [9]. CBT in this context is based on learning theory and uses exposure-based techniques to modify maladaptive responses to negatively valenced stimuli. However, the application of CBT in pediatric dentistry is currently limited in most countries due to constraints in availability, trained personnel, and successful implementation [10].

Internet-based cognitive behavioral therapy (ICBT) was partially developed to address some of the challenges associated with traditional CBT treatments, such as high costs and limited access to trained therapists. Research has previously indicated that ICBT is effective for children with DP [11]. However, the long-term effects of the treatment remain unknown. If ICBT demonstrates positive long-term effects, it has significant potential to enhance availability and cost-effectively assist children and adolescents with dental anxiety in reducing their anxiety and more willingly accepting dental treatment.

The purpose of the study was to assess the long-term efficacy of ICBT on DP, as well as injection phobia (IP), in children and adolescents assessed 1 year after the completion of treatment. The hypothesis was that the effects of ICBT will be maintained at the follow-up assessment.

## Methods

### Study Design

This study was a long-term follow-up, pooling participants from the research group’s pilot study [12] and randomized controlled trial (RCT) [11], which focused on an ICBT for DP and IP. Both studies used the same recruitment strategies, treatment program, and measurements; however, the RCT implemented a randomization process to assign participants to either direct treatment or a waiting list control group after their inclusion and pretreatment measurements. The waiting list control group received the treatment after the 12-week posttreatment measurement. The follow-up assessment was administered 1 year after the completion of treatment.

### Study Setting and Participants

The study was conducted in Sweden, and the intervention was delivered as therapist-guided ICBT via an online platform, with concurrent visits to participants’ regular dental clinics. In Sweden, both general and specialist pediatric dental services are publicly funded and made available at no cost to all children. Routine dental examinations for children and adolescents are scheduled on an annual or a biennial basis. The intervention was delivered independently without direct collaboration with the children’s dental care providers. A central component was parental involvement, wherein parents assumed the role of a coach to support and guide their child or adolescent throughout the treatment. The intervention was therefore designed and targeted for children and adolescents with DP and IP aged 8-15 years. Participants who met the inclusion criteria (Textbox 1) were invited to join the study, and those who met any of the exclusion criteria (Textbox 2) were excluded.

**Inclusion criteria for internet-based cognitive behavioral therapy (ICBT).**

**Inclusion criteria**:

- The child’s age is from 8 to 15 years.
- The child and parents sign informed consent forms.
- A psychologist establishes a diagnosis of dental phobia (DP) or injection phobia (IP) according to the Diagnostic and Statistical Manual of Mental Disorders, Fourth Edition (DSM-4) based on the results of the internet parent version of the Development and Well-Being Assessment (DAWBA) in the initial screening and of the Kiddie Schedule for Affective Disorders and Schizophrenia for School-Age Children – Present and Lifetime (K-SADS-PL) in the semistructured diagnostic interview.
- The child’s and parents’ Swedish language skills are sufficient to manage treatment and the questionnaires.
- Access to a computer and the internet is readily available.
- The child and parents have sufficient time and motivation to work with ICBT 3 hours each week for 12 weeks.
- The parents agree to book at least three visits at the dental clinic during the 12 weeks of treatment.
- If a child is diagnosed with IP, the parents agree to exposure training for intraoral injections at the dental clinic, even if the child does not require dental treatment.

**Exclusion criteria for internet-based cognitive behavioral therapy (ICBT).**

**Exclusion criteria**:

- The child and parents obtain full points on the child and parent versions of the Picture-Guided Behavioral Avoidance Test (PG-BAT). The maximum score of 17 means that the child and the parent assess that the child can already manage the most challenging dental situations.
- The child and parents obtain a score of 31 or less on the child and parent versions of the Children’s Fear Survey Schedule – Dental Subscale (CFSS-DS), and there is no diagnosis of injection phobia (IP) by the psychologist during screening.
- There is a previously established diagnosis of a neurodevelopmental disorder or a likely diagnosis of a neurodevelopmental disorder according to the results of the Development and Well-Being Assessment (DAWBA) or the psychologist during screening.
- Other psychiatric disorders, such as severe depression, eating disorders, or self-harming behaviors, should be treated before addressing specific dental phobia (DP).
- Psychiatric/psychological examination is ongoing or planned.
- Psychological treatment is ongoing or planned.
- The child has had stressful life experiences over the past 12 months, such as parental divorce or physical illness, that the parent or psychologist views as obstacles to treatment.
- The child has undergone cognitive behavioral treatment for dental fear and anxiety (DFA) or DP/IP in the past 3 years.

### Recruitment Process

Participants were recruited through dental clinics and Facebook advertisements, targeting parents of children in the appropriate age range and dental professionals. Information was also shared in Facebook groups directed at dental professionals and psychologists. Dental clinics throughout Sweden were informed about the study and encouraged to promote participation in their waiting areas using posters. The dentists, especially specialists in pediatric dentistry, were asked to recommend patients with severe dental or injection fears that hindered their dental treatment to apply for the study. Interested families were directed to an online platform hosted by the Department of Dental Medicine at Karolinska Institutet. The study’s website offered information about the research and its target population. Interested parents or caregivers could apply directly through the website, after which they would receive login credentials for online screening.

The recruitment period for the pilot were from August 2014 to February 2015 [12], while the recruitment for the RCT spanned from October 2015 to December 2019 [11]. The RCT’s recruitment timeline had to be extended due to slower-than-expected participant enrollment. Please refer to Schibbye et al [11] and Shahnavaz et al [12] for more specific information about each process.

### Screening Procedure

The first stage of the online screening provided detailed information about the study and included a study participation approval form for informed consent for caregivers (hereafter referred to as “parents”). After obtaining informed consent, one parent (designated to complete the questionnaires and participate in the telephone interview) and the child provided basic background information through an online questionnaire. Subsequently, they completed four standardized assessments online: the Picture-Guided Behavioral Avoidance Test (PG-BAT) [12], the Children’s Fear Survey Schedule – Dental Subscale (CFSS-DS) [13], the Children’s Negative Cognitions in Dentistry (CNCD) scale [12], and the Injection Phobia Scale for Children (IPSC) [14].

After evaluating the initial stage of screening, parents of eligible participants were asked to complete the Development and Well-Being Assessment (DAWBA) [15] and were provided with new login credentials to access the assessment on a different platform. A clinical psychologist conducted a telephone interview with the parent using a semistructured diagnostic interview based on the specific phobia section of the Kiddie Schedule for Affective Disorders and Schizophrenia for School-Age Children – Present and Lifetime (K-SADS-PL) [16] to determine whether the child met the criteria for a diagnosis of DP or IP. The psychologist also reviewed the DAWBA results with the parent to identify potential exclusion criteria, which were addressed during the interview.

### Allocation Process

The study began by establishing baseline outcome measurements. In the pilot study, all participants could then start their treatment immediately, while in the RCT, they were randomized to either begin treatment directly or be placed on a 12-week waiting list. After 12 weeks on the waiting list, participants completed the outcome measurements again and were then offered an opportunity to start treatment; thus, the follow-up measurement in the RCT served as the baseline measurement for this group in this study.

### The Treatment

The ICBT implemented in the study was based on a previously published manual [17]. The primary component of the treatment was exposure therapy, delivered through video and audio recordings, a home-based toolkit, and in vivo exposure sessions at a dental clinic. Additional treatment components included psychoeducation, behavioral analysis, controlled breathing techniques, and parental education (Textbox 3). Parents were encouraged to determine who among them would take on the role of coach, responsible for guiding and supporting the child or adolescent throughout the treatment process. A specific treatment module was designed for the coach (week 2) to provide information about effectively supporting, motivating, and assisting the children or adolescents with their assigned tasks. One of the coach’s responsibilities included contacting each participant’s local dental care provider to arrange at least three in vivo exposure sessions conducted by the clinic’s regular dental staff. Participants downloaded instructions for exposure exercises from the platform and either mailed or submitted digital copies to the clinic. In the second week of treatment, participants also received a toolkit containing dental instruments and a Visual Analogue Scale (VAS) designed to facilitate home-based exposure exercises for the children, adolescents, and their coaches.

**Outline of the 12 internet-based cognitive behavioral therapy (ICBT) modules, with 1 module for each week.**

**ICBT modules:**

- Introduction to cognitive behavioral therapy (CBT) and the online treatment
- For the coach only: psychoeducation, practical arrangements, home assignments, how to guide a child to elicit and reinforce behavioral change, reward strategies, and enhancing the child’s self-efficacy
- Behavioral analyses, child psychoeducation and treatment rationale, and goal setting
- Constructing an exposure list and beginning exposure
- Continued exposure (films, training package) and controlled breathing
- Dentistry-related communication training and preparation for the first dental visit
- Evaluation of the dental visit and cognitive restructuring
- Midterm evaluation of ICBT and of exposure at the dentist’s, and relaxation techniques
- Pain and pain management education; fear, thoughts, and pain; and focus shift and acceptance training
- Problem solving and mindfulness training
- Repetition, strategies for maintaining change and relapse prevention, and letter to yourself
- Relapse prevention plan, enhancing self-efficacy, and diploma

The intervention consisted of 12 online treatment modules (Textbox 3), provided to participants at a pace of 1 module per week. Each module included written content, with most containing images, animations, and videos or audio recordings of dental procedures to enhance exposure therapy. Each participant was paired with a specific psychologist for support during the treatment process. In total, 3 psychologists were involved to support the online therapy, all of whom held at least a master’s level (5-year) degree in clinical psychology. Additionally, all had face-to-face clinical experience in CBT for DP and IP in children and adolescents.

Each week, participants and their coaches completed a new module that ended with an assignment made up of knowledge-based questions and practical exercises. After finishing the assignment, participants submitted their answers and a log of their completed tasks to their assigned psychologist. The psychologist provided feedback and granted access to the next module within 2 business days. Participants could also message their psychologist directly via the platform, with responses typically given within 2 business days. In cases of inactivity, psychologists would send reminders through SMS or email to encourage the continuation of the modules. If a participant remained inactive for more than 10 days, the psychologist would attempt to reach out to them by phone.

The intervention and all related data collection took place on a secure online platform under the supervision of the Internetpsykiatri (the Internet Psychiatry Clinic), Stockholm Health Care Services, Region Stockholm, Sweden. Both the coach and the participants used the same login credentials to access the platform. After 12 weeks, all treatment modules were made accessible to participants on the platform, regardless of whether they had completed them. The accounts for the child or adolescent and their coach, along with their login credentials, remained active for an additional 12 months. However, participants could no longer communicate with their psychologist once the treatment period concluded.

### Primary Outcome Measurements

The study used two primary outcome measures. The first was a diagnosis of DP and IP, which was assessed using the specific phobia section of the K-SADS-PL. A clinical psychologist administered this assessment during a semistructured telephone interview with the parent. The K-SADS-PL is a well-established instrument with demonstrated reliability and validity in assessing psychiatric diagnoses [16].

Since it is preferable to use the child as the informant for DFA [18], an additional self-assessed primary outcome was also included, which was the PG-BAT [12]. The PG-BAT was rated independently by both the child and the parent. The PG-BAT assesses the number of dental procedures (n=17; response options: yes=1, no=0) that participants or their parents believe the child can accept and handle voluntarily (Textbox 4). The test features images of 17 dental procedures arranged in a sequence that increases anxiety-provoking potential. Each image includes a written description of the procedure and its context. Scoring ranges from 0 (unable to even enter the dentist’s office) to 17 (capable of managing all dental treatment steps). The two outcomes were both considered primary, reflecting their importance in addressing the participants’ central clinical issue: the ability to accept dental treatment and the functional impairment resulting from the inability to access dental care. Because of this, the designation of primary outcomes from the pre-registration was revised to include the clinician-rated diagnosis originally listed as a secondary outcome, as this collectively offers a more comprehensive assessment of the same underlying construct. This approach incorporates multiple perspectives and informants—including those of the clinician—and better captures the core dimension of the participants’ clinical presentation and functional impairment.

**Dental procedures included in the Picture-Guided Behavioral Avoidance Test (PG-BAT) used in internet-based cognitive behavioral therapy (ICBT).**

**Dental procedures in the PG-BAT:**

- Enter the treatment room.
- Sit in the treatment chair, with a paper bib around the neck.
- Sit in the treatment chair while the chair is lowered.
- Lie in the dental chair, with the lamp turned on and the dental tools close by on a tray.
- Open the mouth and let the dentist look into the mouth.
- Let the dentist use a small saliva ejector in the mouth.
- Let the dentist blow air and water in the mouth.
- Undergo a clinical examination with a dental mirror.
- Undergo a clinical examination with a mirror and a dental probe.
- Let the dentist take an X-ray in the back of the mouth.
- Receive topical anesthesia.
- Receive an injection of a local anesthetic.
- Let the dentist use a large saliva ejector in the mouth.
- Let the dentist attach composite filling to the surface of a tooth.
- Let the dentist drill with a high-speed drill.
- Let the dentist polish a filling with a low-speed drill.
- Let the dentist extract a tooth.

### Secondary Outcome Measurements

The CFSS-DS is a widely used tool for assessing DFA. It consists of 15 items rated on a 5-point Likert scale ranging from 1 (no fear) to 5 (high fear). These items evaluate various situations related to dental and medical care, and ratings are provided separately by both the child and the parent. The Swedish version of the CFSS-DS has shown strong psychometric properties [13].

Children also assessed the 5-item CNCD scale using a 10-point VAS. The endpoints of the scale were 0 (no negative thoughts whatsoever) and 10 (some negative thoughts). These endpoints were visually represented by happy and sad figures, respectively. The CNCD measures the presence and intensity of five common negative thoughts associated with dentistry: feelings of uncontrollability, distrust in dentists, unpredictability, perceived danger, and pain related to dental procedures [12].

The IPSC, completed by the child, consists of 18 items, each assessed on a 5-point Likert scale ranging from 1 (no fear) to 5 (high fear). The items evaluate fear associated with various injection-related situations and procedures. The IPSC has demonstrated strong psychometric properties [14].

Participants also completed the Swedish version of the 14-item Self-Efficacy Questionnaire for Phobic Situations (SEQ-SP), which is evaluated on a 5-point scale ranging from 1 (low self-efficacy) to 5 (high self-efficacy) [19].

Additionally, parents completed the Swedish version of the 12-item Parental Self-Efficacy Questionnaire for Dental Anxiety (P-SEQ-DA) [12], which is assessed on a 10-point scale ranging from 0 (no parental self-efficacy) to 10 (very high parental self-efficacy).

### Assessment Schedule

Pretreatment baseline outcome measurements were collected, which included the PG-BAT and the CFSS-DS, rated separately by both the child and the parent. Additionally, the child completed the CNCD scale, the IPSC, and the SEQ-SP. The parent also filled out the P-SEQ-DA.

The posttreatment measurement was conducted 12 weeks after the treatment start and included all baseline outcome measures and a semistructured diagnostic telephone interview by a clinical psychologist using the specific phobia section of the K-SADS-PL. Additional questionnaires gathered qualitative insights into the child’s current dental anxiety and experiences with ICBT, using a combination of free text, multiple-choice questions, and the VAS. These included questions about adverse events or unintended treatment effects, which were also addressed during the psychologist’s interview.

A follow-up was conducted 1 year after the posttreatment measurement and included all baseline outcome measurements, including the diagnostic telephone interview by a clinical psychologist.

### Statistical Analysis

All statistical analyses were conducted using R version 4.3.2 (R Foundation for Statistical Computing) [20]. A type I error rate of 5% was accepted for all analyses. Before conducting the analyses, the data were assessed for normality. The McNemar test was conducted to analyze the differences in diagnosis (yes or no) between paired observations. ANOVA was performed to evaluate potential differences between baseline, posttreatment (12 weeks), and the 1-year follow-up on continuous outcome measurements. A post hoc analysis using a generalized linear model (GLM) with repeated measures and pairwise comparisons was conducted to estimate effect sizes using Cohen d, which represents the standardized mean difference [21]. A GLM with repeated measures, along with Bonferroni correction for multiple comparisons, was used to analyze predictor effects.

The analysis used an intention-to-treat approach, meaning that all participants who started the treatment were included regardless of the degree to which they completed it. Sensitivity analyses were performed by using maximum likelihood estimation to impute missing values into the dataset.

### Ethical Considerations

The study received approval from the regional ethics review board in Stockholm (ID 2014/633-31/5) and was pre-registered at ClinicalTrials.gov (NCT02588079). Informed consent was obtained before participant enrollment in the study and screening process completion. Study information and consent forms were provided in Swedish, and consent included authorization for the secondary data analysis without needing additional approval. For participants with two caregivers, consent was required from each caregiver separately. No financial compensation was offered to participants, and the intervention was provided at no cost, consistent with the provision of dental care for children and adolescents in Sweden.

## Results

### Participant Details

**Figure 1:**
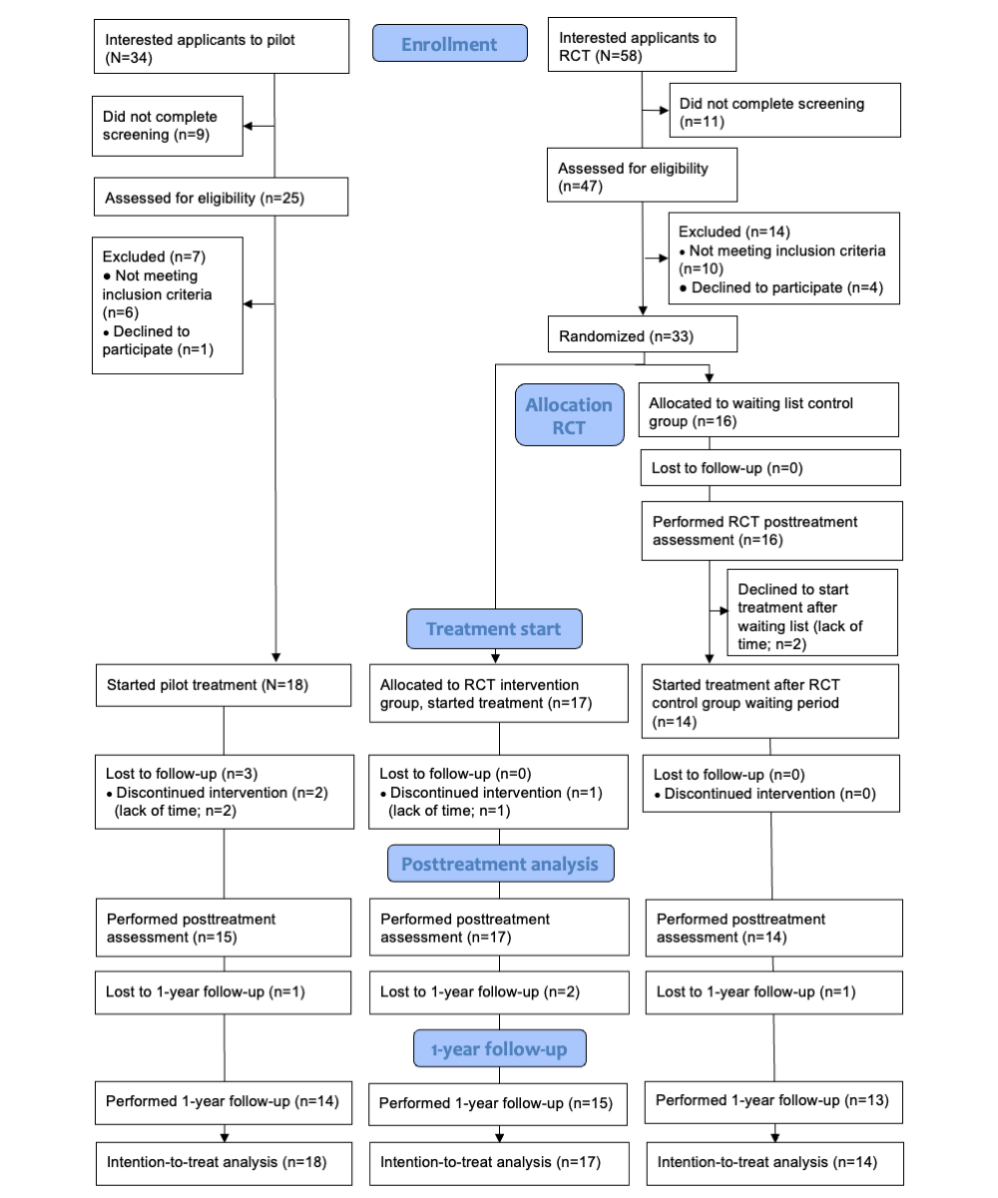
Flowchart of participants in the pilot study and the RCT of ICBT. ICBT: internet-based cognitive behavioral therapy; RCT: randomized controlled trail.

Of the 16 participants in the ICBT control group, 14 (88%) began the treatment after the 12-week waiting period. Those who did not start treatment reported that a lack of time was the reason for their decision. As a result, a total of 31 (94%) participants from the RCT and 18 (100%) participants from the pilot study started ICBT. Consequently, a combined total of 49 participants was included in the long-term analysis of ICBT. For a flowchart of the participants in the ICBT, see Figure 1.

Of the 49 participants who began the treatment, 39 (80%) indicated that their main fear related to dentistry was injections. The majority had experiences with sedation and restraint during previous dental treatments. The most reported specific phobias other than DP and IP among the participants were blood and bee/wasp phobias. For a complete overview of the characteristics of the participants, see Table 1.

**Table 1:** Characteristics of participants in ICBT^a^ from the pilot study and the RCT^b^.

Variables		ICBT pilot sample (n=18)	ICBT RCT sample (n=31)	Total ICBT sample (N=49)
Age (years), mean (SD)		10.9 (2.4)	11.1 (1.9)	11.1 (2.1)
Females, n (%)		12 (67)	20 (65)	32 (65)
Both parents born in Sweden, n (%)		14 (78)	27 (87)	41 (84)
Living with both parents, n (%)		12 (67)	26 (84)	38 (78)
**Duration (months), mean (SD)**				
	Fear of the dentist	47.4 (43.3)	44.8 (42.8)	45.8 (42.5)
	Fear of injections	73.7 (46.7)	67.9 (49.4)	70 (48)
**Diagnosis, n (%)**				
	DP^c^	16 (89)	26 (84)	42 (86)
	IP^d^	17 (94)	27 (87)	44 (90)
	DP and IP	15 (83)	22 (71)	37 (88)
	Another specific phobia^e^	5 (28)	6 (19)	11 (22)
**During previous dental treatment, n (%)**				
	Sedation was administered	8 (44)	29 (94)	37 (76)
	Restraint was used	13 (72)	19 (61)	32 (65)
	General anesthesia was administered	4 (22)	6 (19)	10 (20)

^a^ICBT: internet-based cognitive behavioral therapy.

^b^RCT: randomized controlled trial.

^c^DP: dental phobia.

^d^IP: injection phobia.

^e^Diagnosis of a specific phobia besides DP or IP.

### Treatment Adherence and Dropout Frequency

After the initial 3 weeks, 3 (6%) of 49 participants did not continue due to time constraints and were classified as dropouts. The remaining 46 (94%) participants completed at least the first 5 modules of the treatment and had started the exposure training, while 40 (82%) progressed to at least module 8 and were identified as treatment completers, having finished the home exposure exercises and conducted in vivo exposure at their local dental clinics by the end of the 12-week period. The average number of modules completed was 9.6 (SD 2.7) of 12.

Of the 49 participants who began the treatment, 47 (96%) completed the posttreatment measurement. At the 1-year follow-up, the parents of 42 (86%) participants and 40 (82%) of the children completed follow-up measurements; the parents who completed the measurements also partook in a clinical phone interview. No participants reported any adverse events of the treatment in the follow-up questionnaires or during the clinical phone interviews.

### Main Outcome Measurements

At the 1-year follow-up, 18 (43%) of 42 participants no longer met the diagnostic criteria for either DP or IP, whereas at the posttreatment measurement, 16 (35%) of 46 participants did not fulfill the criteria for either DP or IP. The McNemar test revealed a significant difference compared to before treatment for both groups (*P*<.001); however, the difference was not significant when comparing posttreatment with the 1-year follow-up (*P*=.77). Table 2 presents the intervention results stratified by diagnosis. A statistical difference from before treatment to posttreatment was observed at the 1-year follow-up in all the analyses. However, as seen in Table 2, the proportion of participants meeting the criteria for the respective disorders, any disorder, or both remained stable from posttreatment to the 1-year follow-up. Among the participants who met the diagnostic criteria for DP at baseline, 8 (32%) no longer met the criteria, while 3 (12%) developed a new diagnosis of DP between the posttreatment and 1-year follow-up measurements. Similarly, of those initially diagnosed with IP, 7 (28%) remitted, whereas 5 (20%) developed IP at the 1-year follow-up compared to posttreatment.

**Table 2:** Participants meeting the criteria for diagnoses of DP^a^, IP^b^, either DP or IP, or DP and IP before treatment, posttreatment, and at 1-year follow-up in ICBT^c^.

Diagnosis	Participants, n/N (%)			*P* value (McNemar test)		
	Before treatment	Posttreatment	1-year Follow-up	Before treatment to posttreatment	Before treatment to 1-year follow-up	Posttreatment to 1-year follow-up
DP	42/42 (100)	25/40 (63)	17/36 (47)	<.001^d^	<.001^d^	.23
IP	44/44 (100)	25/41 (61)	20/37 (54)	<.001^d^	<.001^d^	.77
DP or IP	49/49 (100)	30/46 (65)	25/42 (60)	<.001^d^	<.001^d^	.77
DP and IP	37/37 (100)	19/35 (54)	11/31 (35)	<.001^d^	<.001^d^	.23

^a^DP: dental phobia.

^b^IP: injection phobia.

^c^ICBT: internet-based cognitive behavioral therapy.

^d^Significant *P* values.

A repeated-measures ANOVA was conducted to examine changes in the number of dental procedures managed by the participants (self-assessed through the PG-BAT) before treatment, posttreatment, and at the 1-year follow-up. The analysis revealed a significant main effect of time, as assessed by both the child and the parent (see Table 3). The effect size of the post hoc pairwise comparisons was then calculated, which showed large effects (Cohen d≥1.1) at the 1-year follow-up. There was no significant difference between the children from posttreatment to the 1-year follow-up; however, parents reported more dental procedures managed at the 1-year follow-up than posttreatment (Cohen d=0.3, *P*=.04). The pre- to posttreatment measurements were also significant, showing large effect sizes, as rated both by the child and the parent.

**Table 3:** Changes rated by children and parents before treatment, after treatment, and at the 1-year follow-up.

Participant			Time points							*F*_2,96_; *P* value			Within-group effect size, Cohen d (95% CI); *P* value			
			Before treatment, mean (SD; 95% CI); participants (n=49), n (%)		Posttreatment, mean (SD; 95% CI); participants (n=47), n (%)		1-year Follow-up, mean (SD; 95% CI); participants (n=42), n (%)					Before treatment to posttreatment			Before treatment to 1-year follow-up	Posttreatment to 1-year follow-up
**Child**																
	Dental procedures managed^a^	11.7 (3.7; 10.7 to 12.8); 49 (100)		14.9 (2.2; 14.3 to 15.6); 47 (100)		15.0 (1.7; 14.5 to 15.6); 40 (95)		50.1; <.001^b^			1.2 (0.8 to 1.5); <.001^b^			1.1 (0.7 to 1.4); <.001^b^		0.1 (–0.2 to 0.3); .70
	DFA^c^	35.1 (11.1; 31.9 to 38.3); 49 (100)		25.5 (8.7; 23.0 to 28.1); 47 (100)		25.9 (8; 23.3 to 28.5); 40 (95)		41.9; <.001^b^			1.3 (0.9 to 1.6); <.001^b^			1.0 (0.6 to 1.3); <.001^b^		–0.2 (–0.4 to 0.1); .26
	Negative cognitions^d^	24 (12.8; 20.3 to 27.7); 49 (100)		11.1 (11.4; 7.7 to 14.6); 45 (96)		14.3 (10.5; 10.9 to 17.6); 40 (95)		38.8; <.001^b^			1 (0.7 to 1.4); <.001^b^			0.9 (0.6 to 1.2); <.001^b^		–0.4 (–0.7 to –0.1); .006^b^
	Injection fear^e^	42.9 (12.1; 39.4 to 46.4); 49 (100)		33.5 (10.4; 30.4 to 36.6); 46 (98)		34.0 (10.5; 30.6 to 37.3); 40 (95)		27.0; <.001^b^			0.9 (0.6 to 1.3); <.001^b^			0.7 (0.4 to 1.1); <.001^b^		–0.1 (–0.4 to 0.2); .55
	Self-efficacy^f^	27.8 (7.5; 26.6 to 29.9); 49 (100)		44.0 (11; 40.8 to 47.3); 46 (98)		42.3 (11.6; 38.6 to 46.0); 40 (95)		65.9; <.001^b^			1.2 (0.8 to 1.5); <.001^b^			1.1 (0.7 to 1.4); <.001^b^		–0.1 (–0.3 to 0.2); .70
**Parent**																
	Dental procedures managed^a^	11.5 (3.9; 10.4 to 12.6); 49 (100)		14.7 (2.4; 14.0 to 15.4); 47 (100)		15.3 (1.9; 14.7 to 15.9); 42 (100)		52.5; <.001^b^			1.1 (0.8 to 1.5); <.001^b^			1.2 (0.8 to 1.5); <.001^b^		0.3 (0.0 to 0.6); .04^b^
	DFA^c^	35.9 (10.8; 32.8 to 39.0); 49 (100)		24.9 (7.5; 22.6 to 27.1); 47 (100)		24.0 (12.8; 20.3 to 27.7); 42 (100)		60.1; <.001^b^			1.3 (0.9 to 1.7); <.001^b^			1.1 (0.8 to 1.5); <.001^b^		–0.2 (–0.4 to 0.1); .27
	Parental self-efficacy^g^	108.4 (16.3; 103.7 to 113.1); 49 (100)		119.8 (15.8; 115.2 to 124.4); 47 (100)		114.9 (17.3; 109.5 to 120.3); 42 (100)		7.4; <.001^b^			0.8 (0.5 to 1.1); <.001^b^			0.3 (0.0 to 0.6); .04^b^		–0.2 (–0.5 to 0.1); .20

^a^Picture-Guided Behavioral Avoidance Test (PG-BAT; score range: 0-17).

^b^Significant *P* values.

^c^Children’s Fear and Survey Schedule – Dental Subscale (CFSS-DS; score range: 15-75).

^d^Children’s Negative Cognitions in Dentistry (CNCD) scale (score range: 0-50).

^e^Injection Phobia Scale for Children (IFSC; score range: 18-90).

^f^Self-Efficacy Questionnaire for Phobic Situations (SEQ-SP; score range: 14-70).

^g^Parental Self-Efficacy Questionnaire for Dental Anxiety (P-SEQ-DA; score range: 0-132).

### Secondary Outcome Measurements

A repeated-measures ANOVA was conducted, and the effect size of the post hoc pairwise comparisons for the secondary outcome measurements was calculated (see Table 3). The analysis revealed a significant effect of time at the 1-year follow-up on all the secondary outcomes. The effect size was large (Cohen d≥0.8) for most outcomes, except for injection fear (Cohen d=0.7) and parental self-efficacy (Cohen d=0.3). For most outcomes, there was no significant difference between posttreatment and the 1-year follow-up, except for negative cognitions, which increased for the children (indicating that they had more negative cognitions about dentistry) at the 1-year follow-up (Cohen d=–0.4, *P*=.006). A sensitivity analysis was conducted, which showed no change in the statistical significance of any of the main analyses after imputing missing data to the dataset.

### Predictor Analysis

Finally, a predictor analysis was conducted to determine whether background variables (participants’ age and sex) or the number of modules completed influenced the outcome measures. The results revealed that age influenced the clinical diagnosis outcomes, with older participants being less likely to meet the criteria for a clinical diagnosis at later time points (see Table 4). Additionally, an effect for the participants’ sex was found, revealing more favorable results for male participants, particularly at the posttreatment time point, on all measurements. However, only the effects on self-rated injection fear, self-efficacy, and parental-rated DFA were statistically significant. A separate analysis that included both age and sex was also performed, where all the results from the previous analyses remained significant. Furthermore, more completed treatment modules demonstrated a significant association with more favorable scores of self-rated DFA, negative cognitions, and increased self-efficacy.

**Table 4:** Predictor effects on the outcome measurements of ICBT^a^.

Outcome measurements		Age, *F*_*df*_; *P* value	Sex, *F*_*df*_; *P* value	Number of modules completed, *F*_*df*_; *P* value
**Diagnosis**				
	DP^b^	*F*_1,34_=9.22; .005^d^	*F*_1,34_=0.01; .92	*F*_1,34_=2.03; .16
	IP^c^	*F*_1,35_=6.44;.012^d^	*F*_1,35_=2.59; .12	*F*_1,35_=0.52; .48
**Child**				
	Dental procedures managed^e^	*F*_1,38_=2.65; .11	*F*_1,38_=1.04; .38	*F*_1,38_=2.41; .13
	DFA^f,g^	*F*_1,38_=1.30; .26	*F*_1,38_=2.74; .11	*F*_1,38_=4.14; .05^d^
	Negative cognitions^h^	*F*_1,37_=0.48;.49	*F*_1,37_=0.88; .35	*F*_1,37_=4.52; .04^d^
	Injection fear^i^	*F*_1,38_=0.43; .43	*F*_1,38_=7.64; .009^d^	*F*_1,38_=0.16; .69
	Self-efficacy^j^	*F*_1,38_=0.00; .96	*F*_1,38_=5.30; .03^d^	*F*_1,38_=14.55; <.001^d^
**Parent**				
	Dental procedures managed^e^	*F*_1,41_=3.95; .05	*F*_1,41_=0.34; .56	*F*_1,41_=1.31; .26
	DFA^g^	*F*_1,41_=0.01; .91	*F*_1,41_=4.32; .04^d^	*F*_1,41_=1.05; .31

^a^ICBT: internet-based cognitive behavioral therapy.

^b^DP: dental phobia.

^c^IP: injection phobia.

^d^Significant values.

^e^Picture-Guided Behavioral Avoidance Test (PG-BAT).

^f^DFA: dental fear and anxiety.

^g^Children’s Fear and Survey Schedule – Dental Subscale (CFSS-DS).

^h^Children’s Negative Cognitions in Dentistry (CNCD) scale.

^i^Injection Phobia Scale for Children (IFSC).

^j^Self-Efficacy Questionnaire for Phobic Situations (SEQ-SP).

## Discussion

### Principal Findings

At the 1-year follow-up, participants who received ICBT demonstrated significant improvements relative to their pretreatment baseline. Furthermore, the results observed posttreatment were maintained over the follow-up period. After 1 year, 49% of participants no longer met the diagnostic criteria for either DP or IP. Specifically, 53% of participants who initially met the criteria for DP no longer did, and 46% of those who initially met the criteria for IP no longer met the criteria for an IP diagnosis. Additionally, participants self-assessed their ability to manage dental procedure steps at the dentist’s significantly higher, showing a large effect size (Cohen d=1.1). They also, compared to baseline, significantly reduced their fear and anxiety, again with a large effect size (Cohen d=1.0), and exhibited improvements across all outcomes, as reported by both the children and their parents. The treatment appeared to be more beneficial for male participants than females and for older individuals, although these findings reflect associations rather than causal relationships. Additionally, a higher number of completed treatment modules was associated with more favorable outcomes of ICBT.

### Comparison With Previous Research

The long-term effects of ICBT are comparable to that of face-to-face CBT, further supporting the notion that exposure-based CBT treatments are an effective long-term intervention in pediatric dentistry. An RCT of face-to-face CBT conducted by a clinical psychologist revealed within-group effects comparable to this study on the Behavioral Avoidance Test (BAT) and the CFSS-DS [17]. However, at posttreatment and the 1-year follow-up, 64% and 91%, respectively, of the participants in that study no longer met the diagnostic criteria for DP, while 38% and 53%, respectively, did not meet the diagnostic criteria for DP at the different time points in this study. This discrepancy might be attributed to a higher level of engagement in treatment due to the direct interaction with participants or the advantage of using trained personnel delivering the in vivo exposures. Additionally, it may also indicate that the structures surrounding the CBT, such as integration with continued dental care, play a crucial role in achieving favorable long-term results from exposure-based CBT in dentistry. The clinic in the face-to-face CBT RCT had trained personnel (clinical psychologists), established routines, and collaborations for referrals back to local clinics that continued the treatment for the participants, whereas the ICBT lacked all these features. In another RCT [22] where specially trained dentists conducted CBT for intraoral IP, the posttreatment effects on their BAT, CFSS-DS, and an injection phobia scale were comparable to the effects observed in this trial. At the 1-year follow-up, 69% of participants were able to receive intraoral injections at the dentist’s, indicating that they likely would not meet the diagnostic criteria for intraoral IP. In this study, 46% of participants lost their initial diagnosis of IP at the 1-year follow-up. This difference may once again be attributed to not only the specifically trained personnel delivering the exposure but also the stricter focus on intraoral injection exposure treatment and diagnosis in that study [22], while this study also examined participants’ ability to receive injections in health care when establishing the diagnosis of general IP. Finally, a systematic review of the long-term effects of ICBT showed an average long-term symptom improvement of 50% across studies of different conditions [23], consistent with the results of this 1-year follow-up of ICBT for DP and IP, showing that ICBT can provide meaningful long-term effects and should be considered in dentistry. Although research on the stability of diagnosed DP and IP in children and adolescents is limited and needs to be better understood, one study found that stability is lower in younger children under 12 years of age [24]. Notably, the sample in this study had an average duration of 46 months for dentist fear and 70 months for injection fear, and the majority had undergone various prior treatment modalities (76% had received sedation, 20% had received general anesthesia), indicating that ICBT is effective for the group that has stable fears and where conventional methods within pediatric dentistry have been unsuccessful.

Interesting predictor effects of ICBT were observed; however, these results should be interpreted with caution due to the relatively small sample size. Older participants more frequently lost their clinical diagnosis of DP or IP during ICBT. Additionally, although not statistically significant, the trend of the CFSS-DS and the number of dental procedures managed also favored older children. Previous findings on age as a predictor of treatment results for anxiety disorders have been conflicting [25]. However, some suggest that children over 12 years respond better to these treatments [26]. A trend, with some results reaching statistical significance, was observed, indicating that males had a better treatment effect than females, especially at the posttreatment time point. This represents a new finding that has not been reported in previous research on ICBT or psychological anxiety treatments for children and adolescents. Future research is needed to determine whether this is related to the specific conditions of DP and IP or whether these effects are seen in combination with this specific treatment. Nevertheless, DFA and DP have been shown to be more common in females than in males [1], indicating that there could be underlying sex differences affecting both the condition itself and, therefore, possibly its treatment.

### Clinical Implications

This study, along with other research on other psychiatric conditions, indicates that ICBT has robust long-term effects for patients who are willing to receive an internet-based intervention and, in many instances, is comparable to in-person CBT. Given the current lack of availability of evidence-based treatment options for children and adolescents with DP and IP, ICBT should be seriously considered for integration into dental practice. However, the methods for effectively incorporating ICBT into standard dental care remain unexplored. This study shows that ICBT can eliminate the need for specialized staff onsite and can be implemented with minimal training of personnel at regular dental clinics. However, further research on the effects of ICBT with better-trained personnel delivering the in vivo exposure is needed since there is a possibility that this could increase its effect. Moreover, the treatment must be presented in such a way that both personnel and patients recognize its benefits; considering the slow recruitment for this study, ICBT needs to be integrated into local practices. Therefore, trained dental personnel aiding in both treatment delivery and its incorporation into ongoing patient care seems imperative. Finally, it is important also to note that efficacy studies on ICBT are based on participants who actively seek this type of treatment or exhibit strong motivation when invited to participate, which may also disproportionately include individuals with higher socioeconomic resources. This distinction must be carefully considered during implementation to ensure the treatment remains both effective and appropriate. ICBT should not be administered to individuals lacking motivation to engage with the treatment or the resources necessary to complete it.

The participants in this ICBT study can be considered selected due to the screening process and inclusion criteria for research participation, as well as the thoroughness of the treatment provided. Those who withdrew or declined to participate indicated that time constraints were the barrier, suggesting that a less intensive treatment could serve patients with limited time and resources, potentially enhancing its use. In addition, in the posttreatment qualitative evaluation, some participants described the intervention as overly extensive and suggested that it could be delivered in a more condensed format. For example, the RCT conducted on children with intraoral IP achieved comparable results using only five sessions. The number of completed modules in the treatment was primarily linked to the cognitive aspects of dental fear, possibly further indicating that a less extensive treatment could still help with the functional aspects of overcoming the challenge of receiving dental care. In the cases of DP and IP, the impaired oral and general health they cause could arguably be the most important aspect to address, aiming to enable the person to receive needed care willingly. Less extensive ICBTs combined with in vivo exposure provided by trained dental personnel, such as dental assistants or hygienists, seem to be a plausible way to implement a treatment that could reach larger patient groups and maintain efficacy. Importantly, they may also be more feasible for implementation in groups with fewer resources, as they place lower demands on the participants and structures around them. The dental staff delivering the treatment would benefit from training in exposure methods to better assist patients through the ICBT process and serve as a bridge to their ongoing dental care at the clinic. Future research is necessary to establish such treatments’ efficacy and effectiveness and determine which patient groups would benefit most from them. A favorable solution would involve establishing an initial assessment that directs the patient to the appropriate level of treatment. This could include a less extensive ICBT that is standardized and focuses more on exposure to a specific stimulus (primarily injections but also aspects such as the drill, gag reflex, or other physiological sensations that might be the main fear) offered at local dental clinics by trained personnel to help patients overcome their hurdles in receiving dental care. When necessary, a more comprehensive ICBT that also emphasizes cognitive aspects, such as the one used in this study, could be provided to those patients who might require it and are interested. Additionally, the option to consult with a clinical psychologist face-to-face is essential for certain groups, such as individuals with comorbid posttraumatic stress disorder (PTSD), other severe psychiatric conditions, or neuropsychological conditions.

The novel findings indicating that age and sex may influence treatment outcomes are intriguing; however, replication in future studies is necessary to substantiate these effects. Given that DFA and DP have been shown to be more prevalent among females, it is possible that sex-specific adaptations to an intervention may be warranted. Future research should establish whether treatment responses for DP, IP, and intraoral IP differ systematically based on sex. If such differences are confirmed, further investigation into potential variations in the etiology of these phobias could inform the development of tailored treatment approaches that address these sex-specific mechanisms.

Moreover, the predicting effect of age on treatment outcomes further underscores the need to explore the efficacy of a more condensed ICBT protocol. A shorter intervention primarily centered on exposures may be a more pragmatic and effective alternative for younger individuals, minimizing the burden of unnecessarily prolonged treatment, while maintaining therapeutic efficacy. This consideration aligns with evidence suggesting that the stability of blood-injection-injury (BII) phobia diagnoses increases significantly after the age of 12 years [24] and that psychological treatments for anxiety disorders might have a better effect on children aged 12 years and above. Consequently, comprehensive CBT interventions for DP and IP should likely be prioritized for older children and adolescents, where the risk of long-term symptom stabilization and treatment efficacy may be greater.

A final consideration concerns how the delivery and organizational responsibility for exposure-based CBT in dentistry should be strategically addressed in policy planning. In most countries, pediatric dentistry and general pediatric health care are administratively and financially separate, often resulting in higher out-of-pocket costs for dental care. Given that DP and IP are psychiatric conditions with a significant impact on overall health, exposure-based CBT should be integrated into the publicly funded health care systems to enhance accessibility and reduce financial barriers. For ICBT, existing national digital health platforms, where available, offer a scalable and cost-effective avenue for dissemination. However, effective implementation into existing dental care structures requires close collaboration with dental services, where the treatment, with its in vivo exposures, is ultimately to be delivered. This underscores the need for coordinated policy efforts across both general health care and dentistry to address the care of patients with a phobia hindering their dental treatment.

### Strengths and Limitations

A key strength of this study is the inclusion of a clinically relevant sample, with a substantial proportion of participants experiencing persistent functional impairment due to their severe fear. Notably, the vast majority had previously required sedation and physical restraint during dental procedures, underscoring the long-term efficacy of ICBT in enabling these individuals to undergo dental treatment voluntarily. Furthermore, the study used multiple outcome measures assessed by the children themselves, as well as by independent observers, allowing for a comprehensive evaluation of treatment effects across various dimensions of phobic impairment. The strong concordance among these measures further reinforces the effectiveness of ICBT. Additionally, as the intervention was delivered in routine clinical settings by personnel without extensive formal training in CBT, the findings suggest robust external validity and feasibility for implementation in general dental practice. However, careful considerations regarding implementation and how trained personnel could possibly potentiate the effect of the treatment need to be done.

However, certain methodological limitations must also be acknowledged. The clinical interviews conducted to establish the posttreatment and 1-year follow-up diagnoses were not blinded, which may introduce potential bias. Nevertheless, given the consistency of treatment effects across all outcome measures, this limitation is unlikely to have significantly influenced the overall findings. Another limitation is that rather than assessing intraoral IP specifically, this study classified participants based on the broader diagnosis of a general IP in the posttreatment and 1-year follow-up assessments. However, this only attenuated the results, showing the robustness of the treatment effects.

Additionally, the generalizability of the results to populations excluded from this study remains uncertain, potentially limiting the external validity of the findings for certain clinical subgroups and those with limited socioeconomic resources. Furthermore, unmeasured confounding variables could have influenced the predictor analyses, as the study was not originally designed to systematically investigate these aspects. The relatively small sample size may also have constrained statistical power in these analyses, potentially obscuring significant associations where trends toward significance were observed. Despite these limitations, the findings suggest clinically interesting effects that warrant further investigation in future research.

### Conclusion

ICBT for children and adolescents with DP and IP appears to maintain its effects over a 1-year follow-up period. ICBT is associated with long-term reductions in fear and allows the child to willingly accept dental treatment, thus leading to improved dental and general health. This study further supports that ICBT is a method that enhances accessibility to evidence-based, long-term, effective treatment for fear and anxiety in pediatric dentistry.

## Abbreviations

BAT: Behavioral Avoidance Test
CBT: cognitive behavioral therapy
CFSS-DS: Children’s Fear Survey Schedule – Dental Subscale
CNCD: Children’s Negative Cognitions in Dentistry
DAWBA: Development and Well-Being Assessment
DBS: dental behavior support
DFA: dental fear and anxiety
DP: dental phobia
DSM: Diagnostic and Statistical Manual of Mental Disorders
GLM: generalized linear model
ICBT: internet-based cognitive behavioral therapy
IP: injection phobia
IPSC: Injection Phobia Scale for Children
K-SADS-PL: Kiddie Schedule for Affective Disorders and Schizophrenia for School-Age Children – Present and Lifetime
PG-BAT: Picture-Guided Behavioral Avoidance Test
P-SEQ-DA: Parental Self-Efficacy Questionnaire for Dental Anxiety
RCT: randomized controlled trial
SEQ-SP: Self-Efficacy Questionnaire for Phobic Situations
VAS: Visual Analog Scale

## Data Availability

The datasets used and analyzed during this study are available from the corresponding author upon reasonable request.
